# dMM-PBSA: A New HADDOCK Scoring Function for Protein-Peptide Docking

**DOI:** 10.3389/fmolb.2016.00046

**Published:** 2016-08-31

**Authors:** Dimitrios Spiliotopoulos, Panagiotis L. Kastritis, Adrien S. J. Melquiond, Alexandre M. J. J. Bonvin, Giovanna Musco, Walter Rocchia, Andrea Spitaleri

**Affiliations:** ^1^Department of Biochemistry, University of ZürichZürich, Switzerland; ^2^Faculty of Science - Chemistry, Bijvoet Center, Utrecht UniversityUtrecht, Netherlands; ^3^European Molecular Biology Laboratory HeidelbergHeidelberg, Germany; ^4^Biomolecular Nuclear Magnetic Resonance Unit, Ospedale S. RaffaeleMilan, Italy; ^5^CONCEPT Lab, Istituto Italiano di TecnologiaGenoa, Italy

**Keywords:** MM-PBSA, scoring function, binding free energies, haddock, protein-peptide interaction

## Abstract

Molecular-docking programs coupled with suitable scoring functions are now established and very useful tools enabling computational chemists to rapidly screen large chemical databases and thereby to identify promising candidate compounds for further experimental processing. In a broader scenario, predicting binding affinity is one of the most critical and challenging components of computer-aided structure-based drug design. The development of a molecular docking scoring function which in principle could combine both features, namely ranking putative poses and predicting complex affinity, would be of paramount importance. Here, we systematically investigated the performance of the MM-PBSA approach, using two different Poisson–Boltzmann solvers (APBS and DelPhi), in the currently rising field of protein-peptide interactions (PPIs), identifying the correct binding conformations of 19 different protein-peptide complexes and predicting their binding free energies. First, we scored the decoy structures from HADDOCK calculation via the MM-PBSA approach in order to assess the capability of retrieving near-native poses in the best-scoring clusters and of evaluating the corresponding free energies of binding. MM-PBSA behaves well in finding the poses corresponding to the lowest binding free energy, however the built-in HADDOCK score shows a better performance. In order to improve the MM-PBSA-based scoring function, we dampened the MM-PBSA solvation and coulombic terms by 0.2, as proposed in the HADDOCK score and LIE approaches. The new dampened MM-PBSA (dMM-PBSA) outperforms the original MM-PBSA and ranks the decoys structures as the HADDOCK score does. Second, we found a good correlation between the dMM-PBSA and HADDOCK scores for the near-native clusters of each system and the experimental binding energies, respectively. Therefore, we propose a new scoring function, dMM-PBSA, to be used together with the built-in HADDOCK score in the context of protein-peptide docking simulations.

## Introduction

Molecular docking is a computational method that investigates the intermolecular complexes formed between two or more constituent molecules. It comprises the process of generating a model of a complex based on the known three-dimensional structures of its components, i.e., the target (protein, or nucleic acids) and the ligand (a peptide, a protein, a small organic molecule), free or bound to other species (Rognan, 2013). The docking procedure consists in the search for near-native ligand conformations and orientations (usually referred to as docking poses) with respect to a target protein, where the structure of the latter is known or modeled. Fast approximate mathematical expressions (so called scoring functions) are used to rank the docking poses based on estimates of the goodness of the conformations obtained for each putative binder and of the binding affinity estimate of the two interacting partners. Pioneered during the early 1980s (Kuntz et al., 1982), molecular docking is still a field of intensive research, as it represents a fundamental component in many drug discovery programs (Meng et al., 2011) and a primary tool for the virtual screening of large chemical libraries (Kitchen et al., 2004). The typical system considered in docking calculations includes the ligand, the receptor, and the solvent molecules. Because of the enormous number of degrees of freedom associated with the solvent, it is usually neglected in the calculations, or implicitly accounted for in the scoring functions. Despite some valuable improvements in the accuracy and efficiency of the molecular docking algorithms, there are still considerable drawbacks and limitations to face. Among these, the reliability of the scoring functions is probably one of the aspects deserving more attention, since discriminating native pose and obtaining a fair correlation between docking scores and experimental activity data remain difficult tasks. These limitations are responsible for the occurrence of false-positive and false-negative hits in the ranked lists resulting from the screenings performed with standard docking methods. Over the years, since the pioneering work of Kuntz et al. (1982), several scoring functions have been developed (Gilson and Zhou, 2007; Huang et al., 2010; Sarti et al., 2013), based on several terms. Despite empirical scoring functions are still widely used in drug discovery since they are faster and relatively accurate, first-principle methods for ranking decoy structures and for predicting affinity should be considered the first desirable choice in docking scoring stage.

Hence, it is a general opinion that molecular docking results may benefit from post-processing with more accurate tools, able to provide higher accuracy in energy scoring of the putative docked poses. Among several docking approaches, HADDOCK is one of the few computational docking programs that follow a data-driven strategy, using experimental data (generated either via NMR experiments, mutagenesis, or mass spectrometry) as pivotal information to generate docking poses (Dominguez et al., 2003). Moreover, the program allows the receptor to undergo small conformational changes upon association with the ligand, a feature that has been deemed as crucial in simulating the binding process (Spiliotopoulos and Caflisch, 2014). HADDOCK has been applied successfully to a plethora of biomolecular systems (Dominguez et al., 2003). Its reliability is highlighted by the excellent evaluations in the CAPRI experiments (van Dijk et al., 2005) and by the fact that more than 60 structures solved via HADDOCK docking have been deposited in the Protein Data Bank (Berman et al., 2000). Moreover, continuous efforts are devoted to integrate HADDOCK with experimental methodologies (Hennig et al., 2012) and other computational techniques (Kastritis et al., 2014) in order to improve its built-in scoring function. Among several other scoring approaches, Molecular Mechanics Poisson–Boltzmann Surface Area, MM-PBSA, is routinely used to evaluate the strength of the complex formation between protein and ligands. MM-PBSA represents a good trade-off between calculation efficiency and accuracy in binding energy calculations and it has been profitably exploited in virtual design since it allows a ranking in different docking runs and between different ligands (Graves et al., 2008; Venken et al., 2011; Yang et al., 2011; Barakat et al., 2012; Genheden and Ryde, 2015). Although MM-PBSA is one of the most used approximate methods for the estimate of binding free energies, it also presents weaknesses that should not be overlooked. In particular, a source of error can be represented by the entropy contribution, which is often neglected when relative binding free energies of similar molecules are computed. Furthermore, the quality of results depends on different computational factors, including the conformational sampled space, the force field, internal dielectric constant, and the set of atomic radii (Weis et al., 2006). Additional MM-PBSA limitations in the estimation of binding free energies are for highly polar molecules such as DNA and RNA (Kongsted et al., 2009), buried ligands (Singh and Warshel, 2010), and in presence of explicit water molecules that might contribute to the binding free energy (Homeyer and Gohlke, 2012). Therefore, several attempts have been made to improve accuracy and predictivity of the MM-PBSA method acting on the solvation term, including polar and non-polar terms. Expedients such as using different PB solvers (Feig et al., 2004), tuning the grid mesh (Harris et al., 2013), and/or the internal dielectric constant (Singh and Warshel, 2010; Hou et al., 2011; Genheden and Ryde, 2015), including crystallographic and/or specific water molecules (Treesuwan and Hannongbua, 2009; Liu et al., 2013; Maffucci and Contini, 2013), have allowed to successfully use MM-PBSA in the binding free energy calculations (Wang et al., 2001). However, there is still room for improvement to make MM-PBSA more efficient and reliable in the binding free energy calculations in different respects.

Here we focus our investigation on the field of protein-peptide interactions (PPIs), which are gaining large interest in the biological and pharmaceutical research (Scott et al., 2016). In fact, the inhibition of PPIs is of paramount importance in drug discovery and development. The main problem in the PPIs simulation is that the protein-peptide interface is large, shallow, and involving several contacts characterized by being weak, transient and non-specific. Therefore, not all the PPI interface contributes equally to the strength of the binding between the partners. PPIs are rather mediated by hot spots, small regions that give the largest contribution to the binding.

In the present study, we investigated the effectiveness of MM-PBSA on evaluating protein-peptide docked complexes using HADDOCK software in order to consider the possibility of exploiting this relatively fast approach as an additional scoring function. We show in the results section the performance of MM-PBSA as a scoring function for the 19 systems (Results—Section MM-PBSA As Scoring Function for Protein-Peptide Docking) and as binding affinity predictor for the systems where reliable experimental binding affinity data were available (Results—Section Correlation between Experimental Binding Free Energies and Scores).

## Results and discussion

MM-PBSA is an end-point method devised to estimate binding free energy (ΔG_comp_) as the difference of the free energy of the complex and those of the unbound receptor and peptide (Massova and Kollman, 1999). Normally, it is performed from a set of snapshots obtained from Molecular Dynamics simulation (Hou et al., 2011). This method is significantly less computationally demanding than alternatives such as free energy perturbation (FEP) calculations and therefore it represents a possible alternative to FEP for virtual screening of large chemical libraries. It relies on the use of implicit solvent (for the PB part) and it requires energy calculations only on the endpoint (bound/unbound) states whereas other approaches require energy calculation along a reaction coordinate. MM-PBSA has already been used as a scoring function in the past with various outcomes (Kuhn et al., 2005; Thompson et al., 2008; Zhou et al., 2009; Genheden and Ryde, 2015) but to the best of our knowledge this is the first time it has been used for a set of PPIs obtained from docking calculations. Previous studies have shown that MM-PBSA is efficient to identify the correct binding poses and rank small molecules for a specific target (Thompson et al., 2008; Hou et al., 2011; Zhu et al., 2013). However, there is no systematic evaluation of the performance of MM-PBSA in identifying the correct docking poses in the protein-peptide context. As mentioned, the free energy of binding was calculated as the difference in free energy between the product state and the reactants state, that is, between the energy of the protein-peptide complex and the sum of the energies of the protein and the ligand in their unbound forms.

We investigated 19 protein-peptide complexes for which structural and thermodynamic data (binding free energy values ΔG_bind_) were available (Table 1). The final MM-PBSA values are calculated as the sum of two molecular mechanics terms (namely Coulomb and Lennard-Jones), which are calculated by HADDOCK, and two solvation terms, including polar and non-polar solvation contributions, which here were calculated using two different Poisson–Boltzmann equation solvers, APBS (Baker et al., 2001) and DelPhi (Rocchia et al., 2001, 2002) in combination with the NanoShaper program (Decherchi and Rocchia, 2013). We decide to use two different solvers to minimize the MM-PBSA aforementioned weakness and interestingly the binding free energy values from the two solvers were in good agreement in all case studies, indicating that the consistency of the approach. (Figure S1).

**Table 1:** **Complexes investigated**.

**PDB**	**Protein/peptide**	**Ref**.	**K_D_**	**ΔG_bind_**	**Techn**.
1CKA_A:B_	C-Crk N-terminal SH3 domain	1	1.90E–6	−31.84	TF
	C3G peptide				
1D4T_A:B_	T cell signal transduction molecule SAP	2	6.50E–7	−35.26	FP
	Signaling lymphocytic activation molecule				
1MFG_A:B_	Erb-B2 interacting protein	3	5.00E–5	−24.51	ITC
	Erb-B2 carboxyl-terminal fragment				
1PZ5_AB:C_	Antibody SYA/J6	4	4.00E–6	−30.76	ITC
	MDWNMHAA peptide				
1SE0_A:B_	Apoptosis 1 inhibitor	5	7.60E–8	−39.89	ITC
	Cell death protein Grim				
1T4F_M:P_	MDM2	6	8.00E–8	−40.44	F
	Peptidomimetic p53				
1T7R_A:B_	Androgen receptor	7	1.10E–6	−33.96	SPR
	FxxLF motif peptide				
1TW6_B:D_	Baculoviral IAP repeat-containing protein 7	8	3.00E–8	−42.87	FP
	Diablo homolog, mitochondrial				
1W9E_B:S_	Syntenin 1	9	1.00E–3	−17.38	CSP
	TNEFYF peptide				
1X2R_A:B_	Kelch-like ECH-associated protein 1	10	1.81E–7	−38.42	ITC
	Nuclear factor erythroid 2 related factor 2				
2AK5_AB:D_	Rho guanine nucleotide exchange factor 7	11	1.40E–5	−27.01	ITC
	8-residue peptide from CBL-B				
2B9H_A:C_	Mitogen-activated protein kinase FUS3	12	8.00E–8	−40.44	FP
	Serine/threonine-protein kinase STE7				
2CCH_AB:E_	Cell division protein kinase 2/cyclin A2	13	2.03E–8	−43.84	CD
	Cell division control protein 6 homolog				
2FOJ_A:B_	Ubiquitin carboxyl-terminal hydrolase 7	14	2.10E–5	−26.66	TF
	p53 peptide 364-367				
2HO2_A:B_	FE65 WW	15	1.16E–4	−22.28	ITC
	Mena Peptide 10				
2HPL_A:B_	PUB domain of mouse PNGase	16	3.60E–6	−31.02	ITC
	C-terminal of mouse p97/VCP				
2O9V_A:B_	Src homology 3 (SH3) domain	17	2.88E–4	−20.18	F
	Paxillin				
2R7G_A:B_	Retinoblastoma-associated protein	18	9.00E–7	−33.30	ITC
	Early E1A 32 kDa protein				
3D1E_A:P_	DNA polymerase III subunit beta	19	1.42E–6	−33.32	ITC
	decamer from polymerase II C-terminal				

*In each complex, the protein is underlined. The values of K_D_ and ΔG_bind_ are expressed in molar and kJ/mol, respectively. The techniques in the last column are: TF, Tryptophan Fluorescence; FP, Fluorescence Polarization; ITC, Isothermal Titration Calorimetry; F, Fluorescence; SPR, Surface Plasmon Resonance; CSP, Chemical Shift Perturbation; CD, Circular Dichroism*.

In our calculations, we observed that the computed binding free energies were larger than those obtained by experiments (Table 2), an overestimation that has been already observed in other systems. This behavior has been often ascribed to the omission of the entropic contribution, which is an approximation typical of these calculations (Gilson and Zhou, 2007; Spiliotopoulos et al., 2012).

**Table 2:** **Complexes investigated**.

**PDB**	**ΔG_bind_**	**HADDOCK**	**ΔG_comp_**	**dΔG_comp_**	**vdW**	**BSA**
1CKA_A:B_	−31.84	−80.0	−1076.9 (72.9)	−316.2 (15.1)	−91.6	1025.5
1D4T_A:B_	−35.26	−105.8	−987.5 (99.7)	−467.3 (20.1)	−282.9	1710.2
1MFG_A:B_	−24.51	−82.9	−975.4 (57.0)	−369.0 (2.5)	−178.8	1175.0
1PZ5_AB:C_	−30.76	−82.2	−855.4 (50.9)	−406.9 (8.8)	−252.3	1386.2
1SE0_A:B_	−39.89	−101.9	−890.8 (50.7)	−378.4 (17.8)	−213.6	1209.3
1T4F_M:P_	−40.44	−119.2	−748.3 (153.9)	−381.6 (25.4)	−262.9	1522.9
1T7R_A:B_	−33.96	−95.1	−1207.5 (58.5)	−330.1 (13.4)	−76.6	1101.9
1TW6_B:D_	−42.87	−70.8	−469.7 (11.8)	−286.5 (6.2)	−208.2	1007.1
1W9E_B:S_	−17.38	−87.9	−574.4 (106.0)	−253.3 (17.6)	−142.2	966.1
1X2R_A:B_	−38.42	−108.4	−1309.4 (33.2)	−453.8 (17.5)	−179.7	1298.1
2AK5_AB:D_	−27.01	−56.7	−509.7 (58.8)	−225.3 (14.5)	−123.5	850.9
2B9H_A:C_	−40.44	−91.2	−993.9 (56.0)	−450.6 (8.1)	−243.6	1677.7
2CCH_AB:E_	−43.84	−112.1	−1046.7 (68.5)	−410.2 (14.8)	−201.9	1485.3
2FOJ_A:B_	−26.66	−52.71	−622.6 (41.4)	−293.1 (11.0)	−177.5	955.1
2HO2_A:B_	−22.28	−49.7	−208.0 (5.8)	−170.5 (10.0)	−132.9	783.8
2HPL_A:B_	−31.02	−95.1	−1182.5 (25.4)	−353.7 (7.3)	−119.1	862.9
2O9V_A:B_	−20.18	−28.9	−201.3 (24.3)	−140.6 (7.3)	−93.9	830.1
2R7G_A:B_	−33.30	−116.5	−1213.4 (93.9)	−462.7 (18.4)	−226.7	1810.5
3D1E_A:P_	−33.32	−72.2	−662.1 (29.2)	−294.3 (10.4)	−168.5	1072.8
Correlation		0.63	0.49	0.66	0.53	−0.58

*Experimental binding free energy ΔG_bind_, MM-PBSA computational and dMM-PBSA, ΔG_comp_ and dΔG_comp_, respectively, for the system studied. The values are expressed in kJ/mol, except BSA in Å^2^. The values shown between parentheses represent the standard error*.

The constituents of each complex (i.e., protein and peptide) were separated and re-docked using HADDOCK. The HADDOCK scores and MM-PBSA binding free energies corresponding to 200 poses were calculated in each system with the HADDOCK's clustering-based approach. Similarly to HADDOCK score calculation, where coulombic interactions are scaled to a fifth, we also dampened MM-PBSA, i.e., the MM-PBSA energies were calculated multiplying both coulombic and polar solvation terms by 0.2. We will therefore compare three different scoring functions, including HADDOCK built-in, MM-PBSA, and dampened MM-PBSA, which we call dMM-PBSA. The two following sections show the performances of each scoring function in discriminating between the correctly and incorrectly docked peptide poses (Section MM-PBSA As Scoring Function for Protein-Peptide Docking) and in correlating with the experimental ΔG_bind_ through the whole dataset (Section Correlation between Experimental Binding Free Energies and Scores).

### MM-PBSA as scoring function for protein-peptide docking

**Figure 1:**
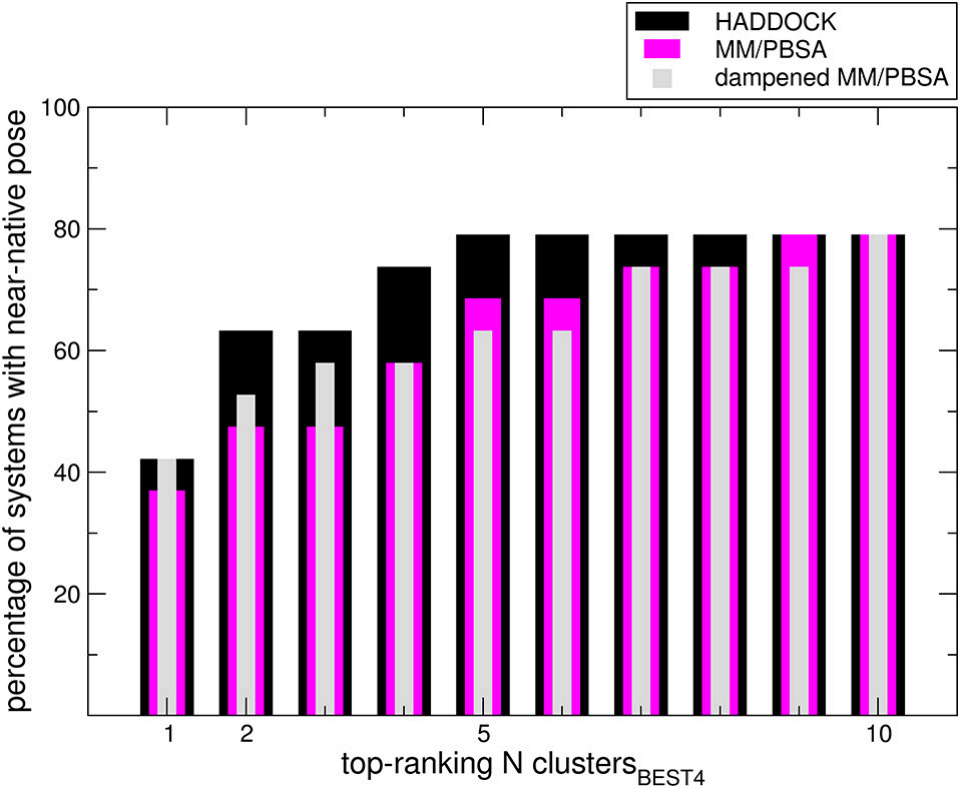
**Bars indicate the percentage of systems in which at least a near-native pose could be found among the members of the N top-ranking (x-axis value) clusters_**BEST4**_**. Note that in four cases no near-native pose could be found among the members of the clusters_BEST4_.

We sought to determine the correlation between the results of the identified scoring methods and the i-RMSD (interface RMSD, see Section Materials and Methods for details) values. The re-docked structures were clustered using the HADDOCK protocol based on i-RMSD values (de Vries et al., 2010). In the Poisson–Boltzmann equation (polar term in MM-PBSA), calculations were performed using ε equal to 2 and 80 for the solute and solvent, respectively. We then calculated the probability to find at least one near-native structure (i.e., displaying an i-RMSD lower than 2 Å) among the *N* top-ranking of the best 4 poses in each cluster (clusters_BEST4_) according to HADDOCK, MM-PBSA, or dampened MM-PBSA (dMM-PBSA), respectively. The percentage of systems with near-native pose vs. the number of clusters for each scoring function is shown in Figure 1. Overall, we observe that HADDOCK score is a valid scoring function by which the near-native pose is ranked within the first cluster in 8 out of 19 systems (about 40%) and within the top 3 clusters in 12 out of 19 (about 63%). MM-PBSA has a somehow worse performance, ranking the near-native pose within the first and the second cluster in 7 out of 19 (about 37%) and 8 out of 19 (about 43%) in the top 3 clusters. The modulation of the polar terms resulting from the MM-PBSA calculation proved able to improve the MM-PBSA performance. In fact, dMM-PBSA reaches similar performance to HADDOCK score in ranking the near-native pose in the first cluster and in the top 3 clusters (11 out of 19, about 58%).

### Correlation between experimental binding free energies and scores

**Figure 2:**
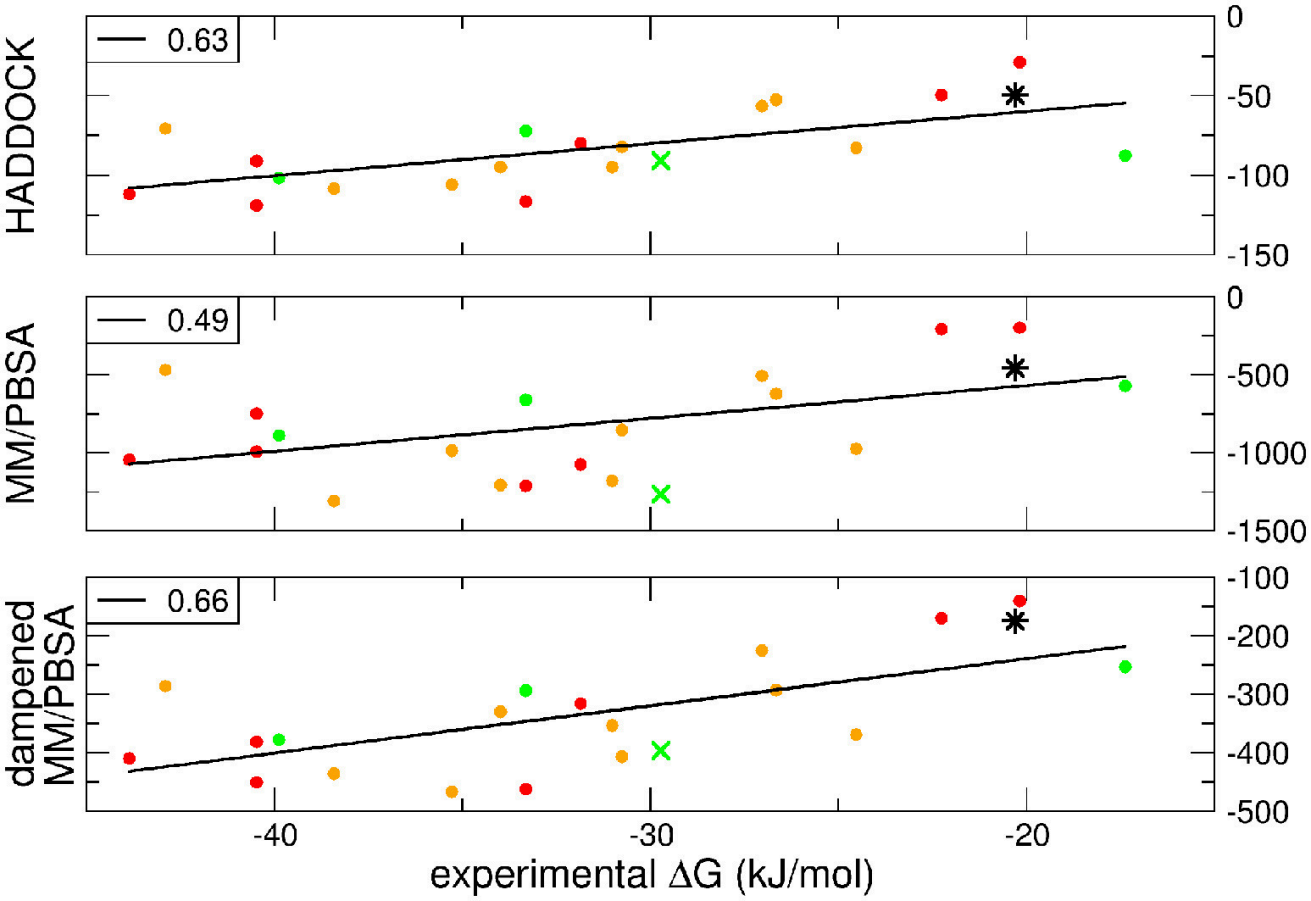
**HADDOCK values are expressed in a.u. MM-PBSA and dampened MM-PBSA values are expressed in kJ/mol**. In all graphs, the color code indicates the average i-RMSD of the cluster_BEST4_. Green, lower than 1.5 Å; orange, between 1.5 and 2 Å; red, >2 Å (none of which is greater than 2.7 Å). Data for AIRE-PHD1 and NPH1-SH3 are indicated with a green × (average i-RMSD: 0.87 Å) and a black star (unknown i-RMSD). The correlation between the different scoring functions and the experimental ΔG_bind_ is shown in the left corner of each panel. The *p*-values for HADDOCK, MM-PBSA, and dMM-PBSA are 0.003, 0.03, and 0.002, respectively.

A further question of interest is whether scoring functions can reliably predict binding affinities when carried out on multiple structures. To address this question, we correlated the HADDOCK scores, MM-PBSA, and dMM-PBSA values of the cluster_BEST4_ displaying the lowest average i-RMSD obtained for each of the 19 systems and plotted against the experimental binding free energies. In Figure 2 it is shown the correlation for each scoring function. Despite the large absolute values, the correlation between the 19 experimental binding free energies and the HADDOCK scores is good (*R* = 0.63 *p* = 0.004, Figure 2, upper panel). The dampened MM-PBSA (Figure 2, lower panel) outperforms MM-PBSA (Figure 2, middle panel) and is better than HADDOCK score in terms of the correlation between experimental and computational binding free energies (*R* = 0.66, *p* = 0.002 and *R* = 0.49, *p* = 0.03, respectively).

We wondered then whether the scores could be exploited to correctly predict the ΔG_comp_ of a new set of protein-peptides. Therefore, we performed a docking with HADDOCK for two additional protein-peptide systems:

1. AIRE-PHD1 complexed with the H3 histone peptide, both NMR structure (PDB 2KE1) and ΔG_bind_ value available (Chignola et al., 2009).
2. NPHP1-SH3 domain in complex with a polyproline peptide, only ΔG_bind_ available (Wodarczyk et al., 2010).

The latter system represents a real blind case study of PPIs since no experimental structural information was available for this system. Binding free energy obtained according to the MM-PBSA approach on the putative pose belonging to the top-ranking cluster_BEST4_ according to the HADDOCK score has been carried out and the calculated value correlated with experimental values (Figure 2, black star). The ΔG_comp_ of the AIRE and NPHP1-SH3 complexes lies close to the previously calculated regression line, suggesting that the scores can be reliably used to predict the correct pose of PPIs systems.

**Figure 3:**
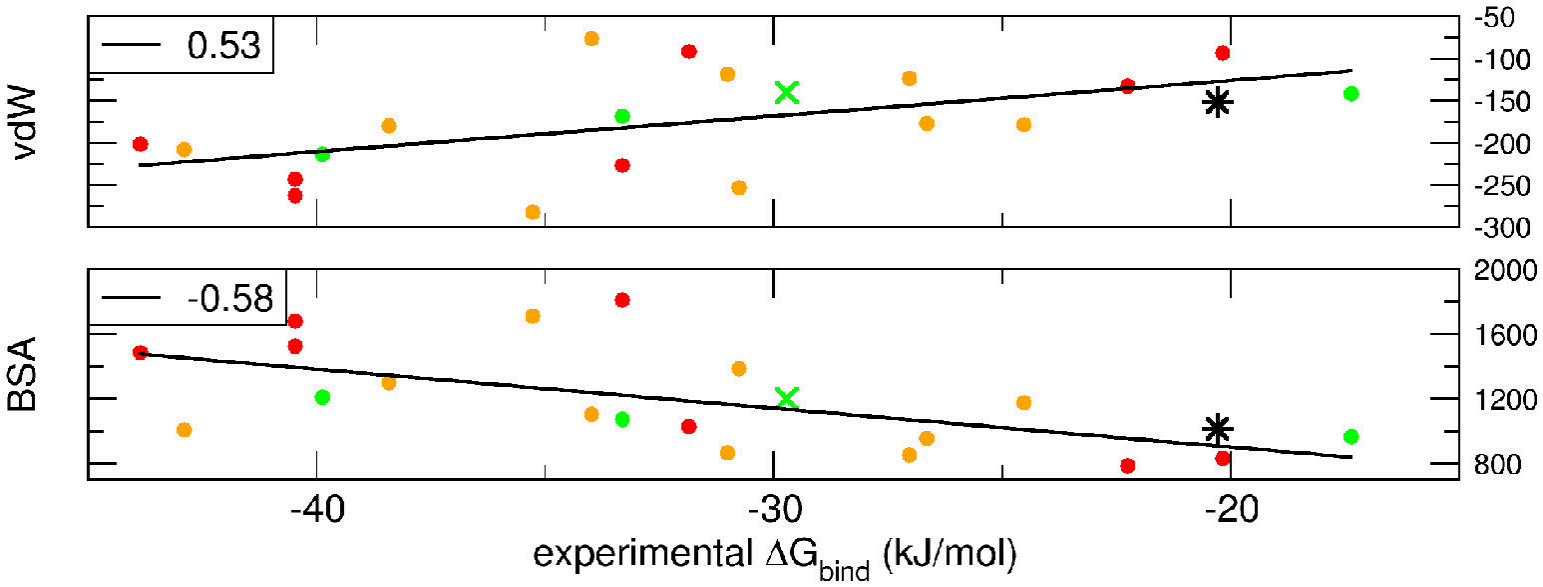
**van der Waals term, expressed in kJ/mol, and BSA, expressed in Å^2^, terms as function of experimental ΔG_**bind**_**. Data for AIRE-PHD1 and NPH1-SH3 are indicated with a green × (average i-RMSD: 0.87 Å) and a black star (unknown i-RMSD). The correlation between the different terms and the experimental ΔG_bind_ is shown in the upper left corner of each panel.

Breakdown of the binding free energy into its components, including van der Waals, electrostatic, polar solvation, and nonpolar solvation interaction energy terms, identified the factors dominating binding affinity for the whole dataset. We analyzed the correlation between either the Lennard-Jones terms (vdW) or the buried surface area (BSA) of the same cluster_BEST4_ and the experimental binding free energies. Data for vdW and BSA vs. experimental binding free energies along with i-RMSD values are plotted in Figure 3. The van der Waals and BSA terms are fairly correlated with the experimental binding free energies (*R* = 0.53, *p* = 0.02, Figure 3, upper panel, and *R* = −0.58, *p* = 0.009, Figure 3, lower panel, respectively). The anticorrelation between experimental ΔG_bind_ values and BSA relies on the fact that larger BSA allows broader interactions between protein and peptide partners. In contrast, no correlation has been found between experimental data and the polar terms.

One of the main advantages to use the MM-PBSA approach in the docking scoring is the ability to quickly perform the computational alanine scanning (CAS) on the best pose in order to evaluate the energetic contribution to the binding affinity of individual residues. Therefore, we carried out a small study of CAS on the AIRE-PHD1 system (five mutants) using the data published in Spiliotopoulos et al. (2012) and we decided to be in the best scenario possible, e.g., we used the NMR complex (Chignola et al., 2009) as starting structure for the CAS calculation. We also evaluated the HADDOCK score of each mutated AIRE-PHD1 complex by only performing the water refinement [i.e., by which the rigid body stage (it0) and the flexible refinement (it1) are turned off]. In agreement with the published data (Spiliotopoulos et al., 2012) we found a good correlation between both MM-PBSA (*R* = 0.88, *p* < 0.02) and dMM-PBSA (*R* = 0.86, *p* < 0.03) and the experimental data (Figure S3 middle and bottom), whereas HADDOCK score showed lower correlation with the experimental data (*R* = 0.60, *p* < 0.21) (Figure S3 top).

## Conclusions

In this work, we made use of the MM-PBSA technique in docking scoring and in affinity prediction of protein-peptide complexes. We also compared the results with the HADDOCK built-in scoring function. Overall, HADDOCK and dMM-PBSA, a dampened MM-PBSA version, behaved similarly in ranking the near-native poses in the top 3 clusters, improving over the standard MM-PBSA version. The introduction of weights for the different MM-PBSA terms is not unprecedented in the literature (Zhou et al., 2009), but this approach has never been applied to PPIs. Notably, despite the fact that different experimental conditions (where the main difference regarded the type of buffer and the ionic strength, whereas as both pH and temperature were comparable) and techniques (Table 1) were used to determine the dissociation constants, we observed a good correlation between experimental and computational ΔG of binding using HADDOCK and dMM-PBSA scoring functions, 0.63 and 0.66 respectively. Interestingly, lack of modulation of the solvation MM-PBSA terms resulted in worse correlation between the experimental and simulated figures (*r* = 0.49).

Our findings were then validated on two additional systems, one with known structure and binding affinity and one for which only the ΔG_bind_ has been reported. Both HADDOCK and dMM-PBSA methods perform remarkably well in ranking the two additional protein-peptide complexes, and lead to good correlation with experimentally measured ΔG_bind_.

In order to assess the presence of possible systematic errors in the binding free energy calculations, we used two different PB solvers, namely APBS (Baker et al., 2001) and DelPhi (Rocchia et al., 2001, 2002). No differences in term of correlation with experimental data were found using the two different solvers, except that in absolute ΔG_comp_ values (Figure S1) and in the calculation speed (see Section Materials and Methods). APBS provided a ΔG_comp_ larger than DelPhi. This could arise from different aspects, including the different approaches used to describe the dielectric interface, the approach used to estimate the reaction field energy, and/or how the different solvers treat cavities that are internal to the solute. The accuracy of the PB equation solution has been reported to be sensitive to the grid size, in favor of smaller grid spacing (Sørensen et al., 2015) However, decreasing the grid spacing increases the computational resources needed to perform the calculation, both in terms of physical memory, and the computational time required. We choose a grid size of 0.5 Å for both solvers since it represents a good trade-off between speed and accuracy. With lower grid resolution, APBS would have encountered problems in convergence in ΔG_comp_ calculation (see Figure 3.2 in Sørensen et al., 2015). Finally, the choice of the interior dielectric (ε_int_) value in the PB calculation is not trivial since in the literature its value can be found spanning between 1 and 20. Higher ε_int_ (4–20) aims to effectively mimic polarization and local rearrangement effects, as well as transient penetration of water molecules into the solute interior. They should in principle be preferred when the PB calculation is performed on individual structures. On the other hand, lower ε_int_ (2–3) is preferred in order to mainly account for electronic polarization and it is commonly used on ensembles of structures, which explicitly account for conformational flexibility. In light of this, there is still no consensus on the most appropriate ε_int_ value. We decided to use ε_int_ = 2 in all cases, while being aware that scaling the polar contribution in dMM-PBSA is similar to considering a dielectric screening of the solute medium, accounting for polarization and rearrangement due to the reaction to the existing fields (Figure S2). This is also in agreement with previous studies where the rank-ordering performance for MM-PBSA improves with increasing dielectric constant (Wang et al., 2013). Simultaneously, this supports the relative importance of the non-polar components and results in better performance. In fact, the van der Waals and BSA terms, which are directly related to the MM-PBSA non-polar terms, correlated well with the experimental data and they provided the main contribution to the final score. These factors might explain the better performance of the dampened MM-PBSA. Finally, we analyzed the calculated data from HADDOCK and MM-PBSA in order to evaluate the reproducibility of the results in terms of scoring and final correlation with the experimental data. First of all, for a given complex, with the corresponding MM energy terms provided by HADDOCK, the only variations in the final results could arise from the PB calculations since it depends on many parameters, including the grid spacing, the atomic radii, and the dielectric interior. For this reason, we used two different PB solvers, APBS and DelPhi, using identical parameters. In Figures S1, S2 it is shown the good agreement in the MM-PBSA and dMM-PBSA calculations with the two PB solvers, indicating that the calculations are quite robust. Second, we calculated the correlations between the experimental and calculated binding free energy using different MM-PBSA values from the cluster_BEST4_ and not the average value. The final correlations R were in a range of 0.39–0.49 for MM-PBSA and 0.60–0.67 for dMM-PBSA, indicating that in principle the MM-PBSA could be performed on a single pose with less computational effort. Finally, the calculated standard error for each cluster_BEST4_ reported in Table 2 represent the MM-PBSA limit when we try to compare the binding affinities of different complexes, mainly when dealing with docking since the conformational sampling is poor with respect to performing MM-PBSA from molecular dynamics simulations (Spiliotopoulos et al., 2012). Nevertheless, in case of AIRE-PHD1 mutants, the MM-PBSA and dMM-PBSA uncertainty over the NMR structure bundle (20 structures) reduces, leading to an average standard error of 34 and 7 kJ/mol, respectively, indicating that dMM-PBSA is fairly reliable in predicting protein-peptide interface alanine mutations. Moreover, the CAS dMM-PBSA of AIRE-PHD1 error range values are fairly in agreement with the CAS MM-PBSA error range (ca. 4 kJ/mol) shown in Spiliotopoulos et al., in which the sampling was carried out by molecular dynamics simulations (Spiliotopoulos et al., 2012). This result indicates that dMM-PBSA carried out on a small number of structures (e.g., 20) behaves similarly to MM-PBSA from molecular dynamics, in which the sampling is more extended. In fact, the modulation of the polar terms in MM-PBSA is probably taking into account the possible local rearrangement similarly to what is done via a higher internal dielectric, indicating that dMM-PBSA represents a simple and promising approach in evaluating alanine mutations from single structure, either from docking or from X-Ray or from NMR.

We believe that in parallel with the recently developed optimization of the HADDOCK score for PPIs inhibitors (Kastritis et al., 2014), the combination of HADDOCK score and modified MM-PBSA binding free energy might lay the groundwork for novel approaches to study *in silico* PPIs inhibitors in a quick and automatic fashion. The advantage of using MM-PBSA as scoring function is threefold. First, MM-PBSA is versatile. It provides estimates of the equilibrium averages over the solvent degrees of freedom, permitting the post-processing of solute representative snapshots from docking poses. Since MM-PBSA estimates binding free energy, it represents a valuable alternative since it is in principle transferable between different docking runs and can be used to score both intra-ligand and inter-ligand poses, saving individual validation for each system under study. This makes MM-PBSA more suitable for novel problems with limited experimental data as we demonstrated in the case study of NPHP1-SH3. Second, MM-PBSA can be used to quantify the thermodynamical strength of the putative poses. In particular, our dMM-PBSA is a reliable scoring function in the protein-peptide field showing good a correlation with experimental data. Therefore, the advantage to use dMM-PBSA with respect to MM-PBSA relies on the possibility to modulate the polar terms without rerunning the Poisson–Boltzmann calculations at different internal dielectric values. Finally, MM-PBSA could better allow to disclose atomistic details of protein-peptide binding, supporting the rational design of bioactive compounds. In fact, post-processing task such as CAS approach has been very recently applied to MD simulations for successfully evaluating the importance of key residues in the protein-peptide binding complex (Spiliotopoulos et al., 2012). Therefore, MM-PBSA can be used to calculate the ΔΔG, defined as ΔG_wt_ − ΔG_mutALA_, on the protein-peptide best docked poses in order to identify residues for which mutation to alanine strongly attenuates binding. The latter behavior occurred in our short CAS study of AIRE-PHD1 mutants (Figure S3), by which an acceptable correlation of *R* = 0.86/0.88 between the experimental and the calculated binding energies of the mutants demonstrated the possibility to use MM-PBSA as a promising tool at low computational cost to evaluate the hot-spots in the PPIs field with respect to the HADDOCK score.

Finally, structural prediction of protein-peptide complexes remains challenging due to two major obstacles: peptides are highly flexible and they often interact weakly with their substrate, underlining their importance in signal transduction or regulation which often relies on transient processes. This leaves flexible docking as one of the few amenable computational techniques to model these complexes (Verkhivker et al., 2000; Hetényi and van der Spoel, 2002; Niv and Weinstein, 2005; Raveh et al., 2010, 2011; Trellet et al., 2013). In our study, we are considering the two interacting partners, protein and peptide, already in the bound conformation, which represents a strong assumption in term of binding mechanism and also the best scenario for a docking calculation, especially in the PPIs field. This undoubtedly increases the success rate in the native pose determination and it reduces the error in the binding affinity calculations since in our MM-PBSA approach we are neglecting most of the solute entropic contribution (i.e., under this assumption the estimate of ΔS = S_Complex_ − (S_protein_ − S_peptide_) can be poor). Recent work have highlighted the efforts to improve HADDOCK protocol in the field of protein-peptide (Trellet et al., 2013) but still predicting large conformational changes remains a challenge as indicated by several failure to accurately predict cases where the protein undergoes large conformational changes upon binding (Trellet et al., 2013). In this case, even the more accurate and reliable scoring function and binding free energy methods will struggle in discriminating the correct binding mode due to both hard and soft docking failure (Verkhivker et al., 2000).

In conclusion, in contrast to other scoring functions and approximate binding free energy calculation methods such as the linear interaction energy (LIE) method, MM-PBSA contains less empirical parameters and, thus, it is more likely to be useful in determining the relative free energies of binding of quite different compounds and systems for which there is more limited experimental data, although a protein structure of the target is, of course, required.

## Materials and methods

### Data set

Particular attention should be paid to the choice of the data set exploited as a benchmark in the binding free energy computational estimation. First of all, binding affinity greatly depends on temperature, pH, and salt concentration (Acampora and Hermans, 1967) and these parameters are difficult to incorporate in the docking calculation. Second, experimental binding data present in the literature are determined with different experimental techniques and they are from different research laboratories. This could significantly impact on the reliability and comparability of the results. Therefore, it should be desirable to rely as much as possible on homogenous data, in terms of research laboratory, experimental technique, and experimental conditions. Along this line, we used a subset of the London's benchmark (http://www.weizmann.ac.il/Organic_Chemistry/London/) for which there were available free forms of the proteins and binding affinity data. The data set consists of 19 complexes the structures of which have been determined by X-ray crystallography, as shown in Table 1. In addition to this data set, two protein-peptide systems, including AIRE-PHD1 (NMR structure, PDB ID 2KE1) (Chignola et al., 2009) and NPHP1-SH3 (Wodarczyk et al., 2010), were used in order to establish the predictive ability of the three different scoring functions. In case of NPHP1-SH3 only experimental binding free energy data were available.

### Generation of binding poses

We used the experimental data (chemical shift mapping and mutagenesis) to generate the decoy structures of the 19 cases study and for both AIRE-PHD and NPHP1-SH3 using the HADDOCK strategy. The HADDOCK protocol proceeds through three steps (rigid docking, semi-flexible docking, and water refinement) (de Vries et al., 2007). Non-bonded interactions were calculated with the OPLS force field using a cutoff of 8.5 Å. The electrostatic energy (E_elec_) was calculated using a shift function while a switching function (between 6.5 and 8.5 Å) was used for the van der Waals energy (E_vdw_). This procedure generated 200 models for each complex, starting from different random velocities. As per default of the HADDOCK protocol, the average score of the top 4 models was considered. The HADDOCK score is defined as a weighted sum of the following four terms:

(1) $$\begin{array}{rcl} \text{HADDOC}{\text{K}}_{\text{SCORE}} & = & 0.2^{*}{\text{E}}_{\text{elec}}\;+\;1.0{\;}^{*}{\text{E}}_{\text{LJ}}\;+\;1.0{\;}^{*}{\text{E}}_{\text{desolvation}}\\ +\;0.1^{*}{\text{E}}_{\text{AIR}} \end{array}$$

where E_elec_ is the electrostatic energy, E_vdw_ is the van der Waals energy, E_desolvation_ is the desolvation energy and E_AIR_ restraints (i.e., distance) violation energies.

The different docking parameter settings and cluster analysis were selected according to the protocol reported in de Vries et al. (2010). BSA is defined as SASA_Complex_ − (SASA_Protein_ + SASA_Peptide_) and it is calculated directly by HADDOCK. All calculations were performed with HADDOCK, version 2.1/CNS, version 1.2, through the refinement interface of the HADDOCK web server (de Vries et al., 2010).

### Binding free energy calculation MM-PBSA

A modified version of the recently published GMXPBSA tool (Paissoni et al., 2014), named HADDOCKPBSA was used to perform the MM-PBSA calculations for the systems. Similarly to the previous version of the scripts, the calculations are organized in an automatic fashion that can be run in parallel in a PBS queue system and the scripts are extensively commented to facilitate their customization. Improving the previous version, HADDOCKPBSA facilitates the interface between HADDOCK and Poisson–Boltzmann Surface Area (PBSA) calculations.

The method for determining the binding free energy following the MM-PBSA approach has been described previously (Massova and Kollman, 1999). The binding free energy of MM-PBSA was estimated as following:

(2) $$\begin{array}{r} <\text{G}>\;=\;<{\text{E}}_{\text{MM}}>+<{\text{G}}_{\text{solv}}>-\text{T}<{\text{S}}_{\text{MM}}> \end{array}$$

This average over each term, i.e., using a set of snapshots, is required since the Poisson–Boltzmann method to calculate G_sol_ averages only over the degrees of freedom of the solvent and not of the solute, i.e., protein, peptide, and ligands.

The energetic term E_MM_ is defined as:

(3) $$\begin{array}{l} {\text{E}}_{\text{MM}}={\text{E}}_{\text{int}}+{\text{E}}_{\text{coul}}+{\text{E}}_{\text{LJ}} \end{array}$$

where E_int_ indicates bond, angle, and torsional angle energies, and E_coul_ and E_LJ_ denote the intramolecular electrostatic and van der Waals energies, respectively. Equation (3) is normally approximated to E_coul_ + E_LJ_ since E_int_ will zero out upon binding (ΔE_int_ = ${\text{E}}_{\text{int}}^{\text{comp}}$ − (${\text{E}}_{\text{int}}^{\text{protein}}$ + ${\text{E}}_{\text{int}}^{\text{peptide}}$)) if the same conformations are considered for the free and bound forms. The solvation term G_solv_ in Equation (4) is split into polar G_polar_ and non-polar contributions, G_nonpolar_:

(4) $$\begin{array}{l} {\text{G}}_{\text{solv}}={\text{G}}_{\text{polar}}+{\text{G}}_{\text{nonpolar}} \end{array}$$

Equation (2) can therefore be rewritten as:

(5) $$\begin{array}{l} <\text{G}>\;=\;<{\text{E}}_{\text{int}}>+<{\text{G}}_{\text{polar}}>+<{\text{G}}_{\text{nonpolar}}> \end{array}$$

where:

(6) $$\begin{array}{r} <{\text{G}}_{\text{polar}}>\;=\;\alpha^{*}\left(<{\text{E}}_{\text{coul}}>\;+\;<{\text{G}}_{\text{polar}}>\right) \end{array}$$

(7) $$\begin{array}{r} <{\text{G}}_{\text{nonpolar}}>\;=\;<{\text{E}}_{\text{LJ}}>+<{\text{G}}_{\text{nonpolar}}> \end{array}$$

where α is a parameter allowing to reduce the polar contribution to the <G> values. Here, the polar contribution G_polar_ was calculated with two different PB solvers: APBS (Adaptive Poisson–Boltzmann Solver) (Baker et al., 2001) and DelPhi (Rocchia et al., 2001, 2002) programs. The polar contribution G_polar_ refers to the energy required to transfer the solute from a continuum medium with a low dielectric constant (ε = 2) to a continuum medium with the dielectric constant of water (ε = 80). G_polar_ was calculated using the nonlinear Poisson Boltzmann equation. The grid spacing was automatically set to 0.5 Å. The temperature was set to 296 K, and the salt concentration was 0.15 M. The non-polar contribution G_nonpolar_ was calculated with two different approaches: APBS internal routine and using the NanoShaper program (Decherchi and Rocchia, 2013). This term was considered proportional to the solvent accessible surface area (SASA):

(8) $$\begin{array}{l} {\text{G}}_{\text{nonpolar}}=\gamma^{*}\text{SASA }+\beta \end{array}$$

where γ = 0.0227 kJ mol^−1^ Å^2^ and β = 0 kJ mol^−1^ (Spiliotopoulos et al., 2012). The dielectric boundary was defined using a probe radius of 1.4 Å.

The binding free energy of a protein molecule to a peptide molecule in a solution was then defined as:

(9) $$\begin{array}{l} \text{Δ}{\text{G}}_{\text{comp}}\;=\;<{\text{G}}_{\text{complex}}>\;-\;\left(<{\text{G}}_{\text{protein}}>+<{\text{G}}_{\text{peptide}}>\right) \end{array}$$

where <G_i_ > is calculated as the average of the best 4 poses of each cluster. The computational determination of the free energy of binding requires the calculation of the entropic contributions to complex formation, including conformational changes in rotational, translational and vibrational degrees of freedom of the solute. Solute entropic contributions are usually estimated by either the quasi-harmonic approach (e.g., Schlitter equation) or by normal mode analysis (Gohlke and Case, 2004). Entropy calculations would require a full sampling of the free energy landscape, an extremely computationally demanding step, which can result in unreliable results (Brown and Muchmore, 2009) with standard errors usually one order of magnitude larger than those associated with the other energetic components (Kar et al., 2011). In addition, the normal mode analysis estimation is often extremely qualitative (Cheatham et al., 1998) and the configuration entropy estimate on a short dynamic time range can be non-significant (Majumdar et al., 2011). Based on these considerations, we decided to neglect the entropic term in our calculations, leading to a one-parameter model:

(10) $$\begin{array}{l} \text{MM-PBSA}\approx \Delta \;{\text{G}}_{\text{comp}}=\alpha^{*}\Delta \;{\text{G}}_{\text{polar}}+\Delta \;{\text{G}}_{\text{nonpolar}} \end{array}$$

where with α = 1 is the canonical MM-PBSA method, α = 0.2 is the new dMM-PBSA method discussed in this work. The standard error (SE) is calculated as follows:

(11) $$\begin{array}{l} \text{SE}=\text{σ}/\surd \text{N} \end{array}$$

where σ is the standard deviation and N is the number of averaged structures (*N* = 4).

Briefly, the protocol obtains the Molecular Mechanics (MM) terms, including intermolecular van der Waals and coulombic terms, for each complex from the HADDOCK output file *energies.disp*. Then, the *get_average.inp* protocol file is modified in order to re-generate the complexes file inserting the partial charges and radii according to the PQR format.

The same PQR files were used for the Poisson–Boltzmann calculations (G_solv_), in order to ensure consistency across the two solvers APBS and DelPhi. Preserving partial charges and intrinsic PB radii is important, as they might significantly affect the outcomes. Notably, each program returns results in different energy units; APBS reports in kJ/mol, and DelPhi reports in kT. All results in this paper are converted to kJ/mol to ease comparison. In APBS six calculations are performed, one for each component in either solvent or “dry” (uniform dielectric ε_ext_ = ε_int_ = 2) environment. The energy (G_solv_) is reported in the “elecEnergy” term and the ΔG_solv_ is then calculated again by subtracting the receptor and ligand from the complex in each environment and then subtracting the values from the dry environment from those of the solvated environment (ε_ext_ = 80). For DelPhi, six calculations are performed, one for each component both in the presence or absence of salt/ions. The energy ΔG_solv_ term used is the difference in “corrected reaction field energy” from the calculations without salt, as well as the difference in the “total grid” energies calculated with and without salt. Surface Area is calculated using the *apbs* built-in function in APBS and NanoShaper functionalities for DelPhi.

Subsequently, the structures of the complex, the protein and the peptide are used to perform the PBSA calculations. HADDOCKPBSA then generates a grid suitable for the calculations for all the structures: the coordinate extremes of the complexes in each dimension are extracted, and 20 and 10 Å are added to each value to set the limits of the coarse and fine grids, respectively. The tool then automatically calculates the number of grid points that is feasible for APBS and DelPhi calculations and builds a mesh finer than 0.5 Å. When all calculations are completed, the final MM-PBSA value is calculated as the sum of the van der Waals and coulombic terms (calculated by HADDOCK) and the polar and non-polar solvation terms (calculated by APBS and DelPhi). HADDOCKPBSA can also extract the HADDOCK scores values and conveniently generate output files that ease the comparison with the MM-PBSA values. HADDOCKPBSA tool is a set of bash script interfacing HADDOCK output with both APBS and DelPhi Poisson–Boltzmann solvers, which need to be installed on their own. HADDOCPBSA is available on the HADDOCK GitHub repository (https://github.com/haddocking).

### Computation time

The PBSA terms were calculated with the two different programs, APBS and DelPhi/NanoShaper. Calculations were carried out on a personal computer with CPU i7 dual-quad core and 16 GB of memory. The time-averaged calculation of the MM part relies on the HADDOCK calculations, which are performed on the clusters. The post-processing time-averaged calculation of the PBSA terms is different depending on the program used. APBS program allows to calculate all-in-once, including PB and SA terms, using the *apbs* tool and it requires ~120 s per complex (about 1000–1500 atoms on average), whereas DelPhi and NanoShaper requires ~12.5 s per complex. Each system comprises 200 putative poses, for a total of 4200 structures analyzed. The performance of the DelPhi solver benefits from the specific approach it uses to estimate the reaction field energy. As described in Rocchia et al. (2001, 2002) the procedure is kept analytical as far as possible. The polarization charge in each grid cube at the boundary between high and low dielectric constant is calculated via Gauss law, then its position is relocated by projecting it over the analytical expression of the Connolly molecular surface. This permits, on one side, to avoid double PBE solution using different dielectric constant values, and, on the other, to get results which are particularly robust regarding position and orientation of the system with respect to the grid. Robustness and efficiency are further enhanced by coupling DelPhi solver with NanoShaper, as shown in Decherchi and Rocchia (2013).

### Statistical treatment of the derived data

Linear correlation between calculated and experimental binding affinities was evaluated via the Pearson product-moment correlation coefficient (R). *p*-values from two-tailed Gaussian probability were determined for each data set using the R, and the sample size information, assuming that correlations are statistically significant if *p* < 0.05. Due to the relatively small size of the samples, we preliminarily performed the Shapiro-Wilk normality test. This test indicated that our data can be modeled according to the normal distribution, (W parameter of 0.96, >0.90, which represents the threshold for 5% significance level). Moreover, the associated *p-*value is 0.63, much greater than 0.05, which is the common accepted threshold to consider a distribution normal. The standard error is calculated as σ/√N, where σ is the standard deviation and N the number of structures (i.e., *N* = 4 for each cluster_BEST4_).

## Author contributions

DS, designed research, ran experiments, analyzed results, wrote the paper; PK designed research, ran experiments, analyzed results; AM designed research, ran experiments, analyzed results; AB designed research, analyzed results; GM designed research, analyzed results, wrote the paper; WR analyzed results, wrote the paper; AS, designed research, ran experiments, analyzed results, wrote the paper.

## Funding

The research leading to these results has received funding under the Horizon 2020 Program, FET-Open: PROSEQO, Grant Agreement n. [687089]. AS acknowledges funding from AIRC (Associazione Italiana Ricerca sul Cancro) through grant MFAG11899. The development of HADDOCK is supported by grants from the Netherlands Organization for Scientific Research (NWO) (TOP-PUNT grant no. 718.015.001) and by European H2020 e-Infrastructure grants (EGI-Engage, grant no. 654142; INDIGO-DataCloud, grant no. 653549; West-Life grant no. 675858 and BioExcel grant no. 675728). The EGI infrastructure and DIRAC4EGI service with the dedicated support of CESNET-MetaCloud, INFN-PADOVA, NCG-INGRID-PT, RAL-LCG2, TW-NCHC, SURFsara and NIKHEF, and the additional support of the national GRID Initiatives of Belgium, France, Italy, Germany, the Netherlands, Poland, Portugal, Spain, UK, South Africa, Malaysia, Taiwan and the US Open Science Grid are acknowledged.

### Conflict of interest statement

The authors declare that the research was conducted in the absence of any commercial or financial relationships that could be construed as a potential conflict of interest.

## Supplementary material

Figure S1 **Representative of MM-PBSA values of the 200 poses of 1CKA system calculated using APBS (x-axis) vs. DelPhi (y-axis) solvers**. The other cases study show similar behavior plot.

Figure S2 **Representative of dMM-PBSA (ε_solute_ = 2 and α = 0.2) values vs. MM-PBSA using ε_solute_ = 5 and α = 1 of the 200 poses of 1CKA system calculated using DelPhi solver**. There is a good correlation between calculating ΔG_comp_ with high ε_solute_ and α, i.e., standard MM-PBSA, and low ε_solute_ and α (dMM-PBSA). Both approaches reduce the ΔG_polar_ term favoring better correlation with experimental data.

Figure S3 **HADDOCK score (top), MM-PBSA (middle), and dMM-PBSA (bottom) calculations of native and mutant AIRE-PHD1/H3K4me0 complexes plotted vs. the experimental binding free energy**.
