# Supplementary figures and images for: dMM-PBSA: A New HADDOCK Scoring Function for Protein-Peptide Docking
Source: Front Mol Biosci. 2016 Aug 31;3:46. doi: 10.3389/fmolb.2016.00046 (PMC5006095; doi:10.3389/fmolb.2016.00046)

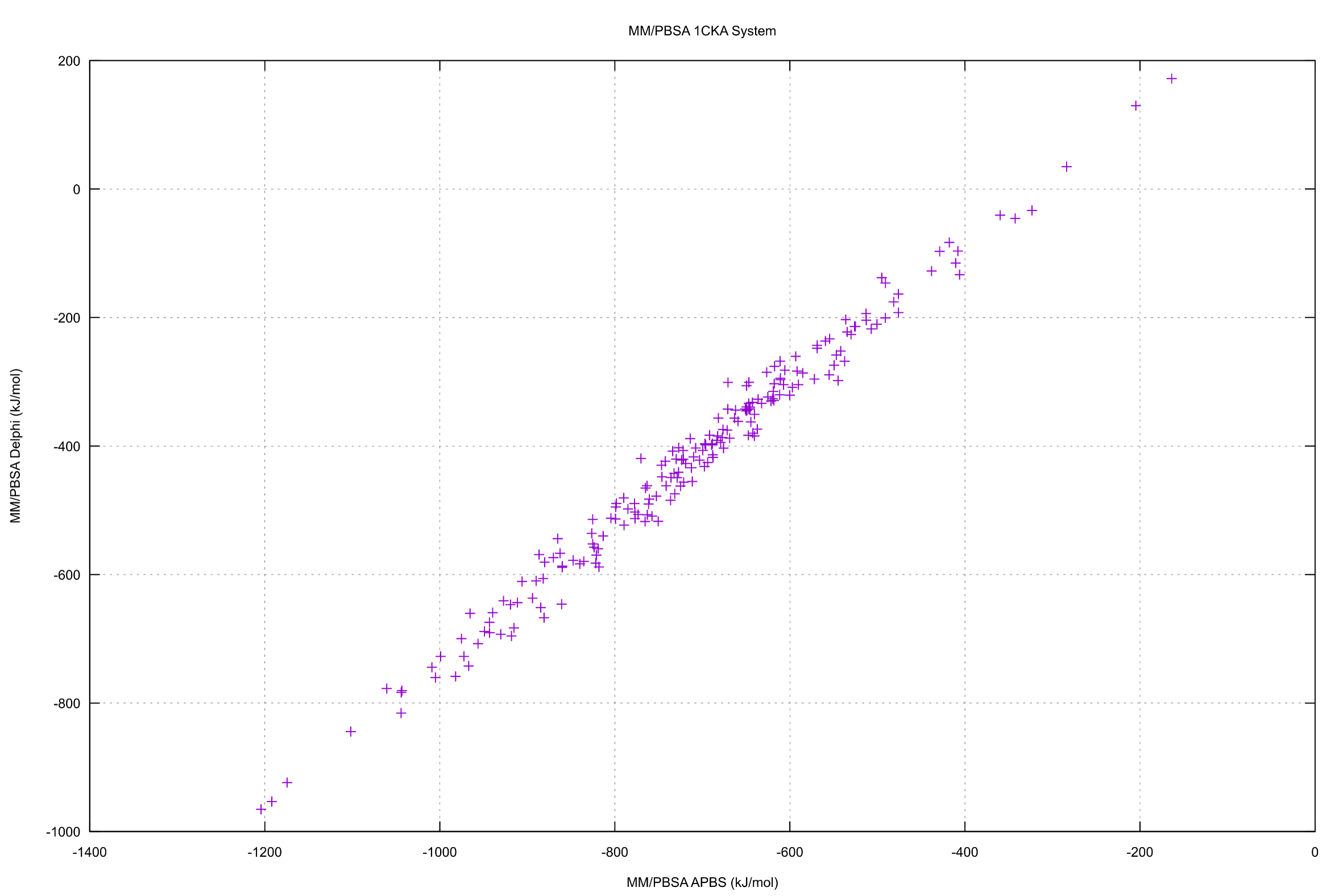

Supplement: Figure S1 — Representative of MM-PBSA values of the 200 poses of 1CKA system calculated using APBS (x-axis) vs. DelPhi (y-axis) solvers. The other cases study show similar behavior plot. [file Image1.TIF]

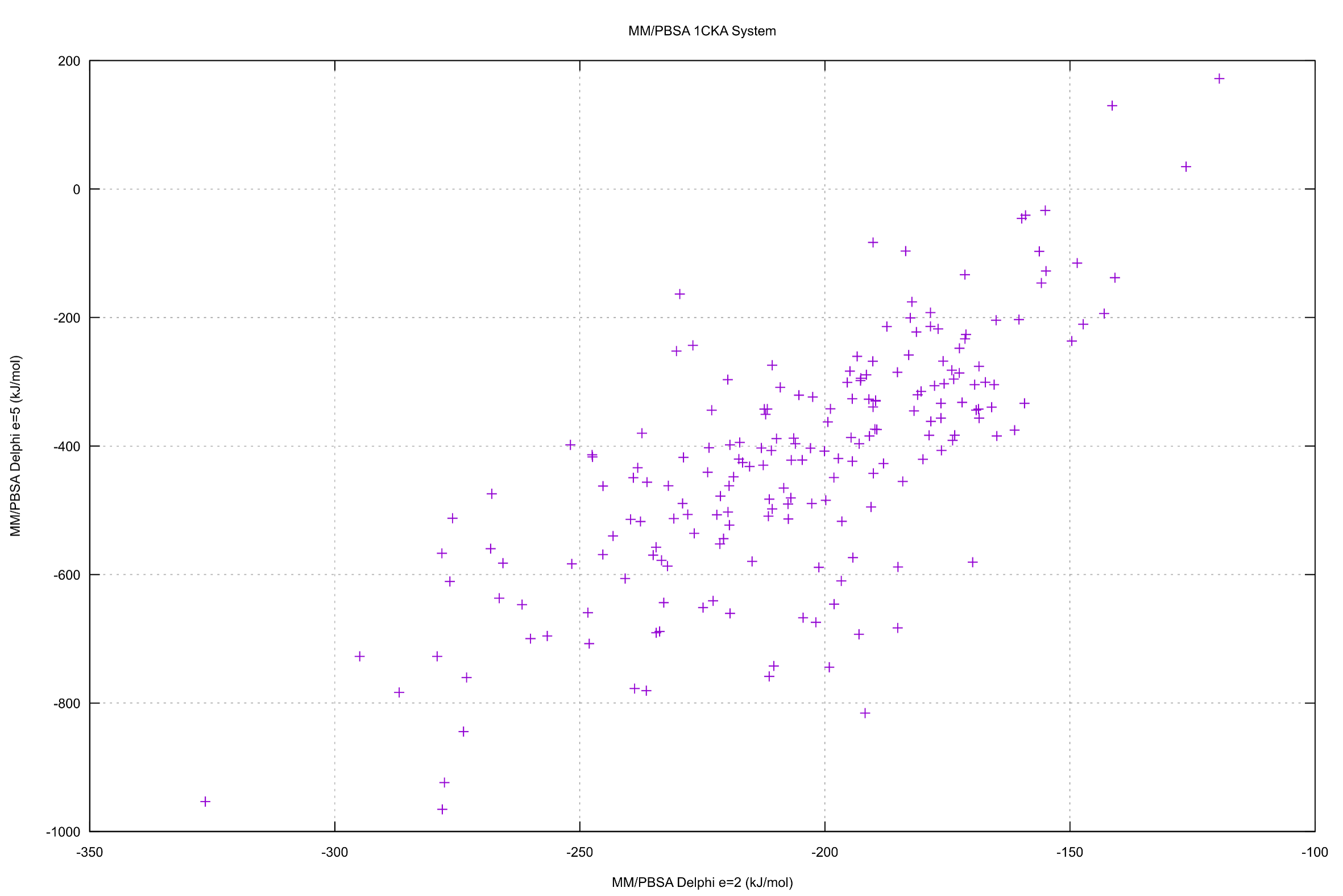

Supplement: Figure S2 — Representative of dMM-PBSA (εsolute = 2 and α = 0.2) values vs. MM-PBSA using εsolute = 5 and α = 1 of the 200 poses of 1CKA system calculated using DelPhi solver. There is a good correlation between calculating ΔGcomp with high εsolute and α, i.e., standard MM-PBSA, and low εsolute and α (dMM-PBSA). Both approaches reduce the ΔGpolar term favoring better correlation with experimental data. [file Image2.TIF]

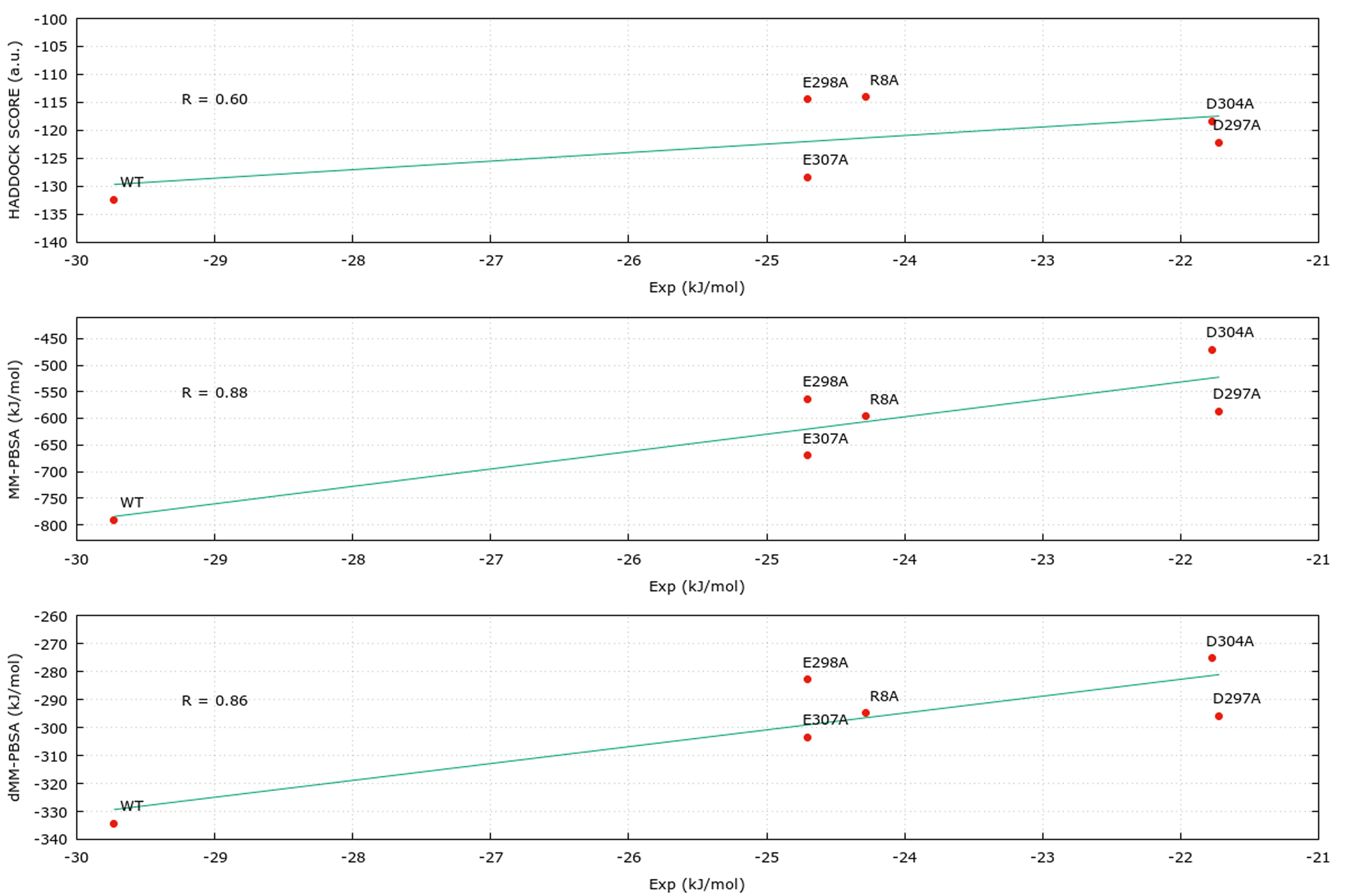

Supplement: Figure S3 — HADDOCK score (top), MM-PBSA (middle), and dMM-PBSA (bottom) calculations of native and mutant AIRE-PHD1/H3K4me0 complexes plotted vs. the experimental binding free energy. [file Image3.TIFF]
